# EPIDEMIOLOGICAL PROFILE OF PATIENTS WITH SYSTEMIC SCLEROSIS TREATED AT A QUATERNARY HOSPITAL IN THE STATE OF SÃO PAULO

**DOI:** 10.1590/1413-785220253302e287081

**Published:** 2025-06-02

**Authors:** Pedro Henrique de Magalhães Tenório, Rafaella Camilo de Oliveira, Matheus Portilho Pinto Ribeiro, Ramon Alves Queiroz, Marcos Felipe Marcatto de Abreu

**Affiliations:** 1 Universidade Estadual de Campinas (UNICAMP), Hospital de Clínicas, Campinas, Sao Paulo, SP, Brazil.

**Keywords:** Epidemiology, Scleroderma, Systemic, Epidemiological Profile, Epidemiologia, Escleroderma Sistêmico, Perfil Epidemiológico

## Abstract

**Objective:**

To report the profile of patients diagnosed with systemic sclerosis and monitored in a four-year hospital in São Paulo.

**Methods:**

Observational study with 51 patients, mostly composed of women. Descriptive statistics such as mean age, proportions (sex, ethnicity, form of the disease), and correlations between functional scores (HAQ, SHAQ, and Rodnan score) were extracted.

**Results:**

The average age found was 49.96 ± 12.01 years. The time since diagnosis was 11.19 ± 10.16 years. When separated by sex, 25% of men presented the limited form, while 65.9% of women presented the localized form. There was a statistically significant correlation (p-value<0.01) between the Rodnan score and the HAQ score, with a value of 0.36 (CI: 0.09-0.58), as well as with the SHAQ score (value of 0.33; CI: 0.05-0.55, p-value=0.01). When evaluating the correlation between the Rodnan score and the Cochin functional scale, there was a statistically significant correlation (p-value<0.01), with a value of 0.38 (0.12-0.58).

**Conclusion:**

According to the literature, this study showed the highest prevalence in women, and the predominant form is limited. ***Level of Evidence II; Cohort Study.***

## RESUMO

**Objetivo:**

Relatar o perfil dos pacientes diagnosticados com esclerose sistêmica e acompanhados em um hospital quartenário paulista.

**Métodos:**

Estudo observacional com 51 pacientes, composto majoritariamente por mulheres. Foram extraídas estatísticas descritivas como idade média, proporções (sexo, etnia, forma da doença) e correlações entre escores funcionais (HAQ, SHAQ e escore de Rodnan).

**Resultados:**

A idade média encontrada foi de 49,96 ± 12,01 anos. O tempo desde o diagnóstico foi de 11,19 ± 10,16 anos. Quando separados por sexo, 25% dos homens apresentaram a forma limitada, enquanto 65,9% das mulheres apresentaram a forma localizada. Houve correlação estatisticamente significante (p-valor<0,01) entre o escore de Rodnan e a pontuação HAQ, com valor de 0,36 (IC: 0,09-0,58), bem como com a pontuação SHAQ (valor de 0,33; IC: 0,05-0,55, p-valor=0.01). Quando avaliada a correlação entre o escore de Rodnan e a escala funcional de Cochin, houve correlação estatisticamente significante (p-valor<0,01), com valor de 0,38 (0,12-0,58).

**Conclusão:**

De acordo com a literatura, este estudo evidenciou a maior prevalência em mulheres e a forma predominante sendo a limitada. ***Nível de Evidência II; Estudo de Coorte.***

## INTRODUCTION

Scleroderma is defined as the presence of thickened and hardened skin (from the Greek "scleros"). This is a distinctive feature of systemic sclerosis (SSc), a chronic disease that affects multiple organs and is characterized by generalized vasculopathy associated with progressive fibrosis of the skin, as well as other organs. Its diagnosis is based mainly on characteristic clinical signs, such as stiffened fingers distal to the metacarpal-phalangeal joints, backed up by serological alterations, such as antinuclear antibodies. SSc is a complex disease with extensive organ involvement, varying progression, and severity, as well as a varied response to surgical procedures.

Previous studies have reported discrepant incidence and prevalence rates. This can be attributed, in part, to the different classifications, geographical discrepancies, and timing of the disease. The global incidence is estimated at between 8 and 56 new cases per million people per year, while the prevalence is estimated at 38 and 341 cases per million people.^1^ Studies report a higher prevalence of SSc in females, with a ratio of affected women to men ranging from 3:1 to 8:1. The disease presents differently based on gender, with women tending to have a more limited form of the disease, an earlier age of onset and a higher frequency of peripheral vasculopathy and risk of pulmonary involvement. In males, there is a higher risk of diffuse skin disease, heart disease, and nephropathy. In addition, the interval between the first episode of Raynaud's phenomenon and the diagnosis of SSc tends to be longer in females.^2^ Patients of African descent tend to have an earlier onset of the disease and more severe forms, with an increased risk of pulmonary fibrosis and renal involvement.^3^

Because of its heterogeneous presentation and evolution, there are different ways of assessing the limitations associated with the disease. The Health Assessment Questionnaire (HAQ) and its version adapted for scleroderma (SHAQ) are widely used tools in the functional and quality of life assessment of patients with rheumatological diseases such as systemic sclerosis. Composed of questions covering various aspects of daily life, the HAQ and SHAQ score the patient's perceived difficulty carrying out everyday activities such as dressing and walking. Its global scores provide a quantitative measure of functional disability and are valuable for clinical assessments and research.^4^ The Rodnan score, or Rodnan Index, plays a crucial role in the clinical assessment of SSc by quantifying the extent of skin fibrosis in 17 specific areas of the body. The scores reflect the severity of skin thickening and are essential for monitoring disease progression and assessing treatment efficacy, contributing to comprehensive clinical management of systemic sclerosis.^5^

This study aims to describe the profile of patients with SSc who are followed up in a São Paulo orthopaedic hospital.

## MATERIALS AND METHODS

An observational study was carried out on 51 patients, mostly women. The average age of the participants was 49.96 ± 12.01 years, with variations between 27 and 76 years. The average time since diagnosis was 11.19 ± 10.16 years, ranging from 1 to 42 years. Patients were diagnosed with systemic sclerosis using the criteria found in the American College of Rheumatology (ACR)/European League Against Rheumatism (EULAR) 2013.^6^ All patients were assessed using the Health Assessment Questionnaire (HAQ), Scleroderma Health Assessment Questionnaire (SHAQ), Cochin functional score and modified Rodnan score. The study was carried out after approval by the institution's research ethics committee (protocol number 23261013.8.0000.5404), under the Declaration of Helsinki. All the data in this study was obtained from the patient's clinical records after informed consent.

### Statistical analysis

The data was statistically analyzed using the R language version 3.1 (R Foundation for Statistical Computing, Vienna, Austria). Quantitative data was characterized using descriptive statistics; qualitative data was characterized using frequency distributions and contingency tables, and comparisons between samples were made using the chi-square test. All P values were bidirectional with a significance level of 0.05%. The statistical correlation used Pearson's method with Yates’ correction, considering p-values of less than 0.05 to be significant.

## RESULTS

The majority of the sample population was made up of women (92.2%), and the most prevalent self-declared ethnicities were white (64.7%), brown (27.5%) and black (7.8%). The patients were classified into 2 types of disease: Limited form (62.7%) and diffuse form (37.3%). (Table 1)

**Table 1:** Frequency distribution of the sample by gender.

Sex	Absolute frequency	Relative Frequency
Female	47	92.2%
Male	4	7.8%
Total	51	100%

**Figure 1:**
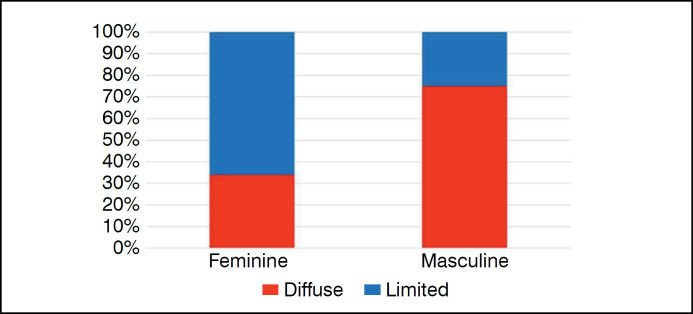
Distribution of forms of the disease by gender.

When separated by gender, 25% of the men (1 patient) had the limited form, while 75% had the diffuse form (3 patients). In the group of women, 65.9% had the localized form (31 patients), while 34% had the diffuse form (16 patients). There was no statistically significant association between gender and the form of the disease (p-value=0.27). (Figure 1)

**Figure 2:**
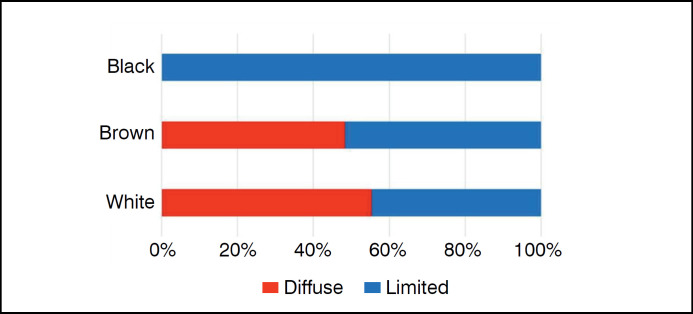
Distribution of forms of the disease, by self-declared ethnicity.

When separated by self-declared ethnicity, 100% of black patients had the limited form (4 patients). In brown people, the prevalence of the limited form was 64.3% (9 patients) and the diffuse form was 35.7% (5 patients). In whites, 57.6% of patients had the limited form (19 patients) and 42.4% had the diffuse form (14 patients). However, these differences were not statistically significant (p-value=0.25). (Table 2 and Figure 2).

**Table 2:** Frequency distribution of the sample by self-declared ethnicity.

Ethnicity	Absolute frequency	Relative Frequency
White	33	64.7%
Brown	14	27.5%
Black	4	7.8%
Total	51	100%

There was a statistically significant correlation (p-value<0.01) between the Rodnan score and the HAQ score, with a value of 0.36 (CI: 0.09-0.58), as well as with the SHAQ score (value of 0.33; CI: 0.05-0.55, p-value=0.01). When the correlation between the Rodnan score and the Cochin functional scale was evaluated, there was a statistically significant correlation (p-value <0.01), with a value of 0.38 (0.12-0.58). (Table 3).

**Table 3:** Frequency distribution of the sample by form of the disease.

Form	Absolute frequency	Relative Frequency
Limited	32	62.7%
Diffuse	19	37.3%
Total	51	100%

## DISCUSSION

This observational study reports on the epidemiological profile of patients with systemic sclerosis followed up as outpatients at a hospital in the state of São Paulo. The relatively high prevalence justifies the small number of patients recruited for the study. According to Horimoto et al. (2017),^7^ the prevalence in a Brazilian municipality (Campo Grande/MS) was 105.6/million inhabitants. SSc is a rare disease, but its incidence has slightly increased in recent years.^8^

In this study, most patients were women, which aligns with the literature. Only 1 in 5 patients with systemic sclerosis is estimated to be male.^9^

The limited form was more prevalent, similar to other results found in the literature. In a multicenter European study, Della Rosa et al. (2001) showed the prevalence of 2/3 of the cases in its limited form.^10^ Similarly, Jacobsen et al. (1998) reported that three-quarters of patients had a diffuse presentation of the disease in a study of Danish patients.^11^ Similarly, Nagy and Czirjak (1997) diagnosed the limited form as the most prevalent in a study carried out in Hungary.^12^ However, these results are not unanimous. Steen et al. (1988) and Bobeica et al. (2021) identified the diffuse form as the most prevalent in epidemiological studies.^9,13^

The limited form was the most prevalent in female patients, while the diffuse form was the most prevalent in men. However, no statistical significance was observed for these differences, which could be explained by the low number of men selected in the sample. The same rationale can be used for the prevalence of the limited form in blacks: although 100% of the patients in the study had this form, the number of participants was too low to be statistically significant. Morgan et al. (2017) showed contrasting differences in the severity and time of manifestation of symptoms when comparing an African-American cohort with European studies.^3^ However, given the low number of patients who self-declared as black, the present study could not show such differences.

When the statistical correlations were analyzed, a weak correlation was observed between the Rodnan score, HAQ, and SHAQ. These measures, in general, limit patients’ quality of life. Thus, despite using different questions, the increase in limitation observed in one questionnaire is expected to be reflected in the others, which was evidenced, albeit weakly.

## CONCLUSION

This study describes the epidemiological profile of systemic sclerosis in a hospital in São Paulo. Similar to what has been found in other publications, the study showed a higher prevalence in women, and the predominant form is limited. The most prevalent form of the disease was limited, especially in women. When stratified by self-declared ethnicity, the most prevalent form in blacks, browns, and whites was limited, with no statistically significant difference. Due to the disease's low incidence and high heterogeneity, multicentre epidemiological studies are necessary for a better characterization and prognosis.
